# Supplementary material for: Non-bonded force field model with advanced restrained electrostatic potential charges (RESP2)
Source: Commun Chem. 2020 Apr 3;3:44. doi: 10.1038/s42004-020-0291-4 (PMC8204736; doi:10.1038/s42004-020-0291-4)
Supplement: Supplementary file 2 — Supplementary Information [file 42004_2020_291_MOESM2_ESM.pdf]

Supplementary Information:
Non-bonded force field model with advanced restrained electrostatic potential charges (RESP2)

1. Dipole Moments

Supplementary Table 1: Molecular dipole moments (Debye) calculated from FF point charges and from QM electron densities. Mean indicates average of the gas-phase and hydrated dipole moments.

	FF point-charges				QM			
		RESP2			HF/ 6-31G*	PW6B95/ aug-cc-pVD(+d)Z		
SMILES	RESP1	gas	δ=0.5	water	gas	gas	mean	water
CCOCC	1.41	1.22	1.47	1.71	1.27	1.07	1.35	1.63
CC1=CC=CC=C1	0.28	0.41	0.40	0.38	0.27	0.40	0.39	0.38
OC	2.16	1.93	2.09	2.25	1.90	1.64	1.85	2.05
COCCC	1.51	1.29	1.48	1.67	1.30	1.06	1.30	1.54
CCCN	1.74	1.53	1.74	1.96	1.47	1.26	1.51	1.75
C-C#N	4.08	4.03	4.21	4.39	4.07	4.03	4.22	4.40
CC(C)CC	0.07	0.10	0.11	0.12	0.07	0.10	0.12	0.13
Cc1ccc(C)cc1	0.00	0.00	0.00	0.00	0.00	0.00	0.00	0.00
CC(=O)C	3.28	3.13	3.58	4.04	3.22	3.07	3.59	4.11
c1cocc1	0.79	0.61	0.64	0.68	0.81	0.64	0.67	0.70
N1(C)CCOCC1	1.46	1.28	1.49	1.71	1.45	1.29	1.50	1.71
c1ccccc1N	1.67	1.66	1.70	1.74	1.59	1.57	1.62	1.67
CN	1.90	1.68	1.86	2.04	1.62	1.37	1.61	1.84
CCCCO	1.89	1.70	1.90	2.11	1.72	1.53	1.78	2.03
CC(C)O	2.09	1.94	2.14	2.34	1.77	1.62	1.91	2.19
C1CCOC1	2.02	1.83	2.12	2.41	2.01	1.81	2.11	2.41
CN(C)C=O	4.46	4.39	4.96	5.54	4.07	4.03	4.71	5.39
CCC#N	4.19	4.15	4.72	5.29	4.17	4.14	4.73	5.32
C1CCCCC1	0.00	0.00	0.00	0.00	0.00	0.00	0.00	0.00

C(CO)O	2.01	1.82	2.07	2.32	1.79	1.59	1.89	2.19
CC(=O)CC1=CC=CC=C1	3.05	2.87	3.40	3.92	2.91	2.73	3.26	3.78
C(=O)N	4.19	3.96	4.53	5.10	4.19	3.95	4.53	5.11
C=CC=O	3.73	3.38	3.92	4.46	3.65	3.37	3.95	4.52
C1COCO1	1.35	1.17	1.35	1.53	1.36	1.18	1.37	1.55
CC(=O)OC(=O)C	4.35	4.03	4.65	5.28	4.42	4.10	4.77	5.43
C(CO)CO	3.71	3.32	3.75	4.17	3.40	3.01	3.50	3.99
CCC(=O)CC	2.99	2.80	3.38	3.96	2.95	2.77	3.38	3.99
CC1=CC=CC=C1C	0.47	0.70	0.89	1.08	0.45	0.68	0.88	1.07
CCCCCC	0.00	0.00	0.00	0.00	0.00	0.00	0.00	0.00
CCC(CC)O	1.96	1.81	2.08	2.35	1.74	1.58	1.91	2.24
NCCO	1.50	1.29	1.42	1.55	1.26	1.09	1.22	1.35
CCCCCCC	0.03	0.04	0.07	0.10	0.07	0.07	0.12	0.17
CCCCCCCCCC	0.00	0.00	0.00	0.00	0.00	0.00	0.00	0.00
CCCCCCCC	0.00	0.00	0.00	0.00	0.00	0.00	0.00	0.00
c1ccccc1	0.00	0.00	0.00	0.00	0.00	0.00	0.00	0.00
CCCCNCCCC	1.46	1.35	1.51	1.68	0.98	0.87	1.12	1.37
OCCCCO	0.00	0.00	0.00	0.00	0.00	0.00	0.00	0.00
OCC(O)CO	4.29	3.85	4.35	4.85	4.03	3.58	4.15	4.72
C1CCNCC1	1.30	1.16	1.44	1.72	1.35	1.23	1.52	1.81
OCCCCCO	3.71	3.31	3.75	4.18	3.35	2.96	3.43	3.90
CC(=O)c1ccc(C)cc1	3.87	3.74	3.86	3.97	3.64	3.53	3.69	3.85
CNCCCN	3.11	2.80	3.16	3.52	2.54	2.17	2.67	3.17
CN(C)CCCN	1.61	1.41	1.61	1.81	1.33	1.16	1.40	1.63
CCN(CC)CCCN	1.67	1.52	1.75	1.98	1.54	1.38	1.63	1.87
CCn1ccnc1	4.26	4.25	5.01	5.76	4.27	4.29	5.06	5.83
CN=C=O	3.18	2.95	3.33	3.69	3.14	2.92	3.29	3.65
OCC#CCO	0.98	0.87	1.02	1.17	1.05	0.95	0.98	1.01
N#Cc1ccco1	5.14	4.81	5.63	6.45	5.15	4.84	5.67	6.49
Cc1cccnn1	4.26	4.01	4.84	5.67	4.31	4.10	5.03	5.95
O=Cc1ccoc1	3.02	2.96	3.45	3.93	2.89	2.89	3.41	3.93
Cc1ccc(C)n1C	2.12	2.22	2.36	2.50	2.07	2.18	2.32	2.46
CC(=O)c1ccco1	3.67	3.53	3.78	4.03	3.49	3.32	3.57	3.81
CN1CCN(C)CC1	0.00	0.00	0.00	0.00	0.00	0.00	0.00	0.00
OCCCCCCO	0.00	0.00	0.00	0.00	0.00	0.00	0.00	0.00
c1cn2ccnc2cn1	3.84	3.72	4.57	5.42	3.83	3.73	4.60	5.46

CCCCn1ccnc1	4.36	4.40	5.11	5.82	4.42	4.49	5.21	5.93
CC(=O)CCC=C(C)C	3.03	2.80	3.32	3.83	2.98	2.75	3.30	3.84
c1ccc2ccccc2c1	0.00	0.00	0.00	0.00	0.00	0.00	0.00	0.00
c1ccc2nccnc2c1	0.27	0.50	0.63	0.76	0.26	0.50	0.63	0.76
OCCCCCCCO	3.34	2.93	3.42	3.90	3.35	2.96	3.46	3.96
CC(C)(C)c1ccncc1	2.85	2.89	3.46	4.02	2.85	2.91	3.49	4.06
Cc1ccc2c(c1)OCO2	0.85	0.67	0.73	0.79	0.65	0.43	0.52	0.61
CCCCC(=O)CCCC	2.95	2.76	3.40	4.05	2.85	2.67	3.37	4.07
O=C1CCc2ccccc2C1	3.31	2.98	3.38	3.78	3.27	2.95	3.36	3.77
OCCCCCCCCO	0.00	0.00	0.00	0.00	0.00	0.00	0.00	0.00
CC(C)(O)CCC(C)(C)O	0.00	0.00	0.00	0.00	0.00	0.00	0.00	0.00
NCCNCCNCCN	3.08	2.75	2.98	3.20	2.30	1.94	2.30	2.65

2. Per molecule error analysis

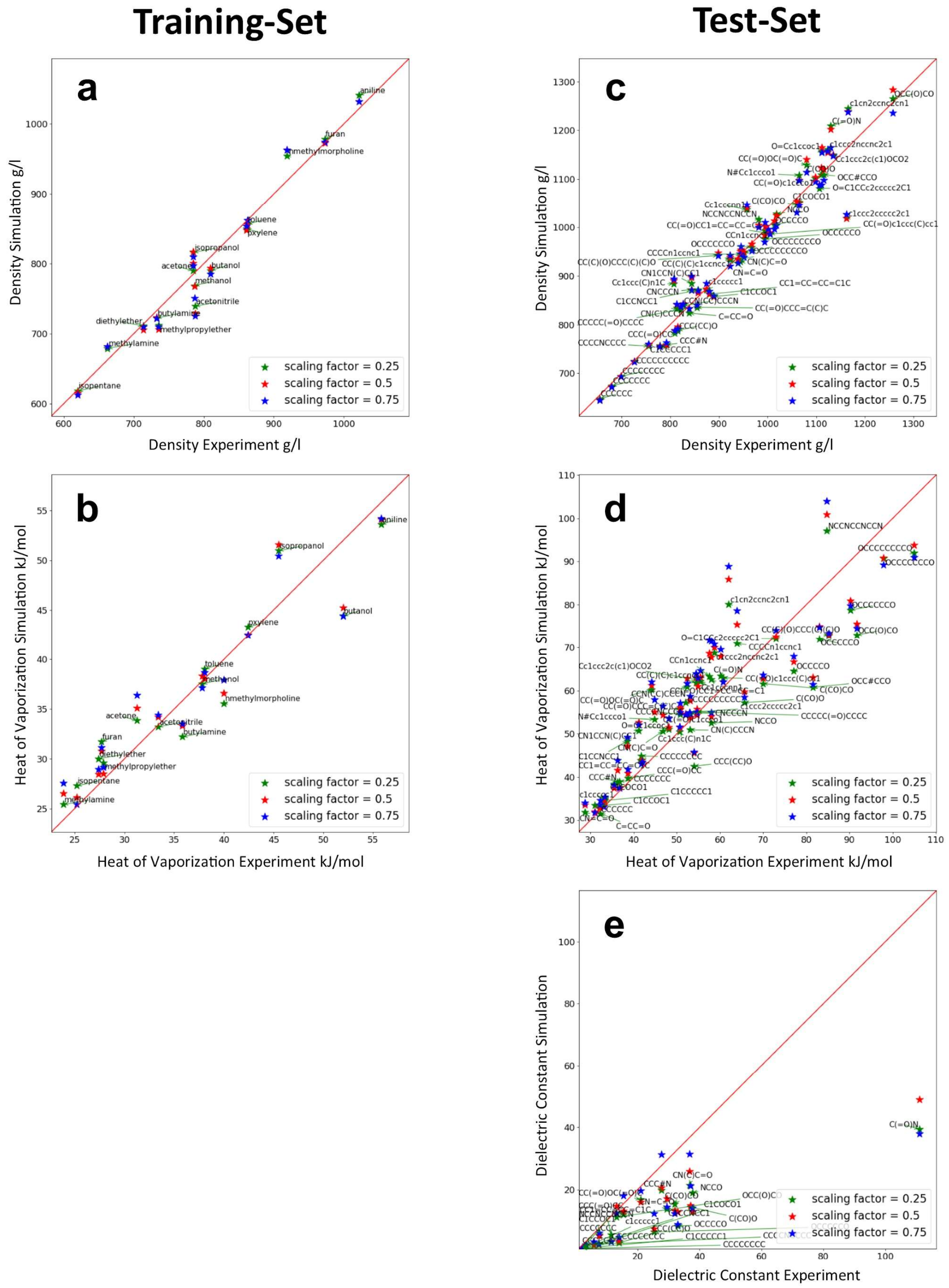

Supplementary Figure 1: Comparison of experimental and simulated results per molecule. *Results for the training-set (A,B) and the test-set(C-E) are shown. Densities are shown in A and C, Heats of vaporization in B and D, and dielectric constants in E.*

3. Point charge dipole moments with RESP2$_{0.50}$

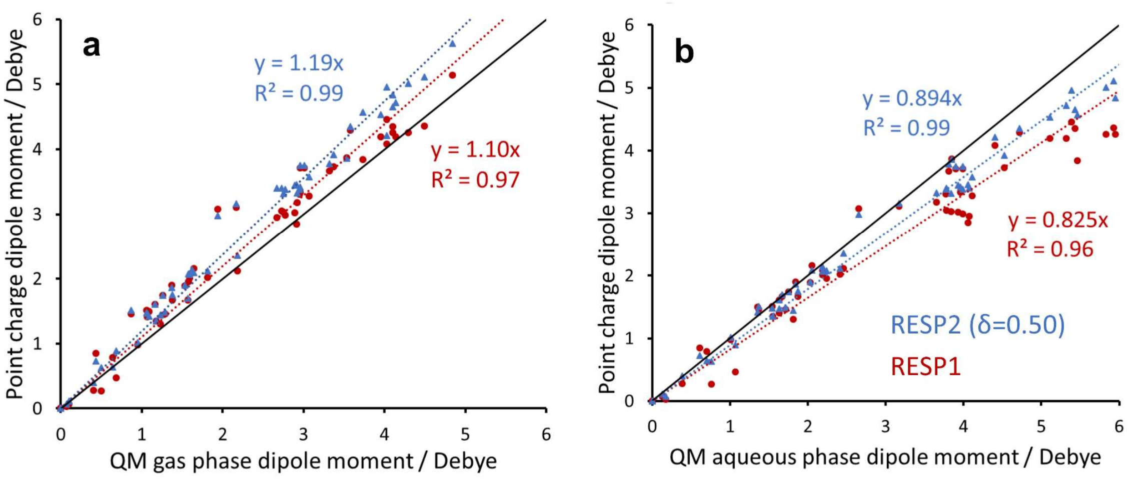

Supplementary Figure 2: Comparison of molecular dipoles moments: *Scatter plots of molecular dipole moments based on RESP1 and RESP2$_{0.50}$ charges plotted against the dipole moments from the gas-phase and aqueous QM calculation.*

4. Molecules used for quantum method benchmarking

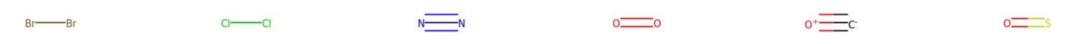

BrBr	ClCl	N#N	O=O	[C-]#[O+]	O=S

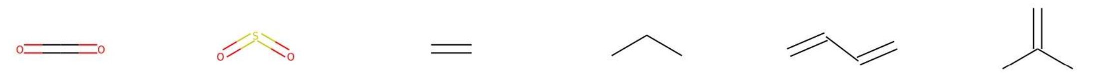

C(=O)=O	O=S=O	C=C	CCC	C=CC=C	CC(=C)C

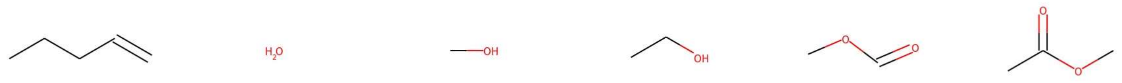

CCCC=C	O	CO	CCO	COC=O	CC(=O)OC

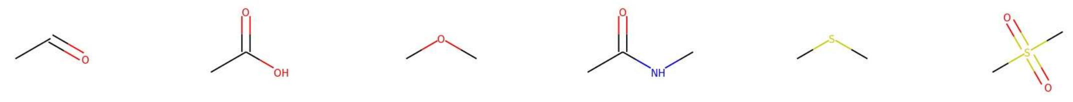

CC=O	CC(=O)O	COC	CC(=O)NC	CSC	CS(=O)(=O)C

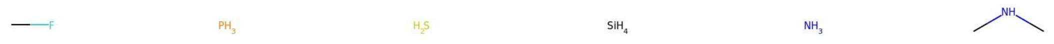

CF	P	S	[SiH4]	N	CNC

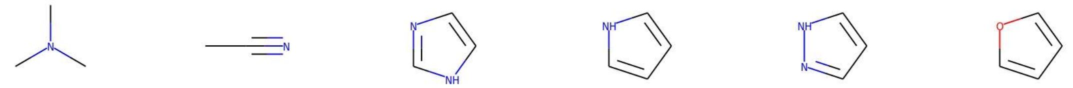

CN(C)C	CC#N	C1=CN=CN1	C1=CNC=C1	C1=CNN=C1	C1=COC=C1

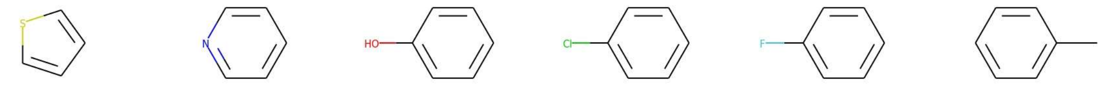

C1=CSC=C1	C1=CC=NC=C1	C1=CC=C(C=C1)O	c1ccc(cc1)Cl	c1ccc(cc1)F	Cc1ccccc1

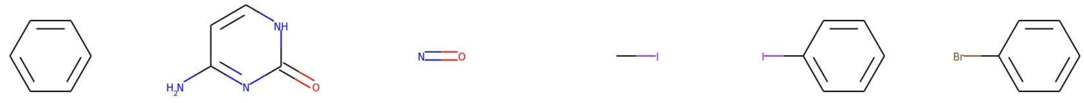

c1ccccc1	Nc1cc[nH]c(=O)n1	[N]=O	CI	c1ccc(cc1)I	c1ccc(cc1)Br

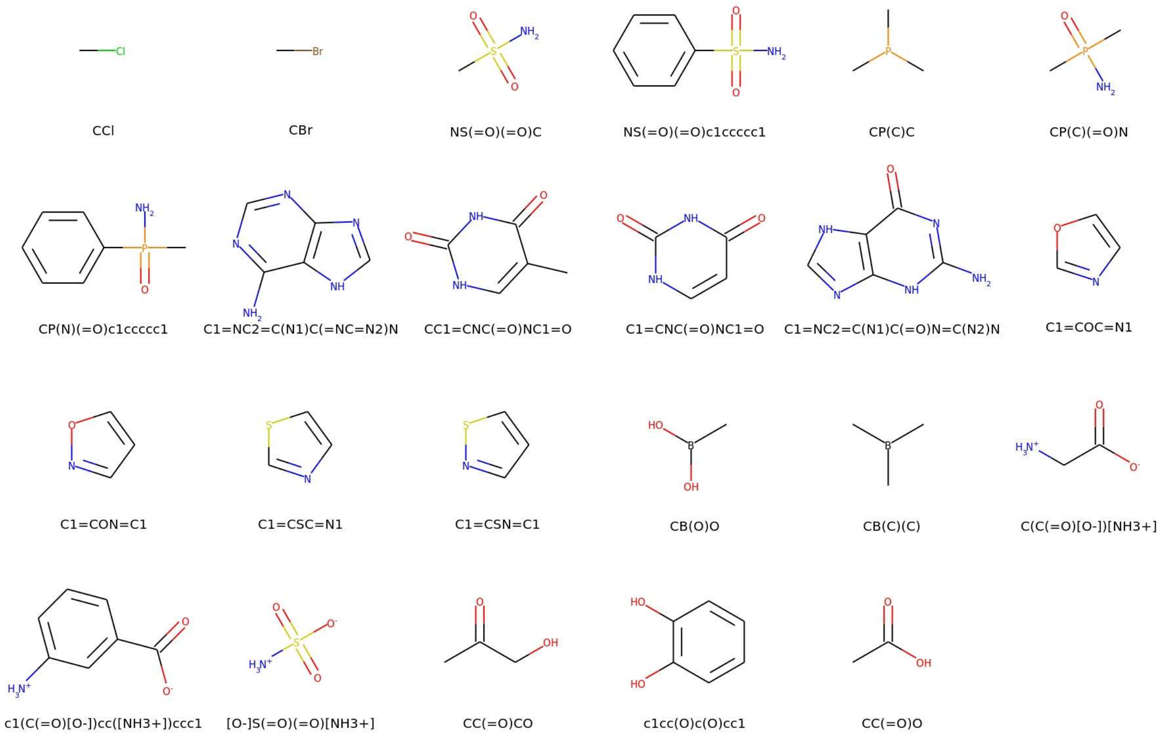

Supplementary Figure 3: Molecules for QM benchmarking: *71 molecules used for the benchmarking of different QM methods to a double-hybrid reference calculation*

5. Starting parameters for the LJ optimization

Supplementary Note 1: Starting parameters for the LJ optimization. Epsilon values in kcal/mol and rmin_half in Å.

<Atom smirks="[#1:1]" epsilon="0.0157" id="n1" rmin_half="1.4870" parameterize="rmin_half, epsilon"/>

<Atom smirks="[#6:1]" epsilon="0.1094" id="n2" rmin_half="1.9080" parameterize="rmin_half, epsilon"/>

<Atom smirks="[#8:1]" epsilon="0.1700" id="n3" rmin_half="1.6837" parameterize="rmin_half, epsilon"/>

<Atom smirks="[#7:1]" epsilon="0.1700" id="n20" rmin_half="1.8240" parameterize="rmin_half, epsilon"/>

<Atom smirks="[#1:1]-[#7,#8,#9,#16,#17,#35]" epsilon="5.27e-05" id="n8" rmin_half="0.300" parameterize="rmin_half, epsilon"/>

6. Experimental values

Supplementary Table 2: Experimental values used for training.

Training Data		
SMILES	Density (g/mol)	Heat of Vaporization (kJ/mol)
CCOCC	713	27.4
CC1=CC=CC=C1	862	38.1
OC	787	37.8
COCCC	736	27.9
CCCN	733	35.8
C-C#N	787	33.4
CC(C)CC	620	25.2
Cc1ccc(C)cc1	861	42.4
CC(=O)C	785	31.3
c1cocc1	973	27.7
N1(C)CCOCC1	919	40.0
c1ccccc1N	1022	55.8
CN	662	23.9
CCCCO	810	52.0
CC(C)O	785	45.5

Supplementary Table 3: Experimental values used for testing.

Test Data				
SMILES	Density (g/mol)	Heat of Vaporization (kJ/mol)	Dielectric Constant	HFE (kJ/mol)
CCCCCCC	680	36.7	1.9	11.2
CCCCCCCCCC	726	52.1	2.0	13.2
CCCCCCCC	699	41.8	2.0	12.0
c1ccccc1	874	33.3	2.3	-3.8
CCCCNCCCC	756	50.8	3.2	-13.6
CCCCN(CCCC)CCCC	774	62.7	2.3	-
OCCCCO	1014	77.1	32.9	-
CCCCCCCCCCCCCCCC	770	81.3	2.1	-
OCC(O)CO	1257	91.7	13.2	-56.2
C1CCNCC1	857	38.6	5.8	-21.4
CCCCCCCCN(CCCCCCCC)CCCCCCCC	807	100.1	-	-
OCCCCCO	994	83.0	25.3	-
CC(=O)c1ccc(C)cc1	1005	60.7	-	-19.7
CN(C)C(=N)N(C)C	913	49.9	23.1	-
CNCCCN	844	53.1	-	-
CN(C)CCCN	813	53.1	-	-
CCN(CC)CCCN	826	52.4	-	-
CCCCCCCCCCCCCCCCCCCCCCCC	799	121.9	2.1	-
CCCCCCCCCCCCCCCCCCCCCC	794	111.9	2.0	-
CCn1ccnc1	995	57.5	-	-
CN(C)CCCN(C)C	779	53.1	-	-
CN=C=O	923	28.7	29.4	-
OCC#CCO	1115	81.5	-	-
N#Cc1ccco1	1065	44.8	-	-
Cc1cccnn1	957	58.0	-	-
O=Cc1ccoc1	1111	48.1	-	-
Cc1ccc(C)n1C	807	50.7	-	-
CC(=O)c1ccco1	1098	53.0	-	-
CN1CCN(C)CC1	844	41.2	-	-31.7
OCCCCCCO	967	90.2	-	-
c1cn2ccnc2cn1	1165	62.0	-	-
CCCCn1ccnc1	945	63.9	-	-

CC(=O)CCC=C(C)C	855	50.8	-	-
c1ccc2ccccc2c1	1162	54.6	-	-10.0
NCCN1CCNCC1	985	68.7	-	-
c1ccc2nccnc2c1	1124	58.7	-	-
OCCCCCCCO	951	97.9	-	-
CC(C)(C)c1ccncc1	923	54.4	-	-
Cc1ccc2c(c1)OCO2	1135	54.9	-	-
CCCCC(=O)CCCC	822	54.7	-	-11.0
O=C1CCc2ccccc2C1	1106	73.0	-	-
OCCCCCCCCO	939	104.9	-	-
CC(C)(O)CCC(C)(C)O	898	85.2	-	-
NCCNCCNCCN	982	84.7	11.4	-
CCOCC	-	-	-	-6.7
CC1=CC=CC=C1	-	-	-	-3.8
OC	-	-	-	-21.3
COCCC	-	-	-	-6.9
CCCN	-	-	-	-18.4
C-C#N	-	-	-	-16.2
CC(C)CC	-	-	-	10.0
Cc1ccc(C)cc1	-	-	-	-3.3
CC(=O)C	-	-	-	-15.9
c1cocc1	-	-	-	-
N1(C)CCOCC1	-	-	-	-26.4
c1ccccc1N	-	-	-	-23.0
CN	-	-	-	-19.0
CCCCO	-	-	-	-19.7
CC(C)O	-	-	-	-19.8

7. Mean signed errors as function of mixing parameter δ

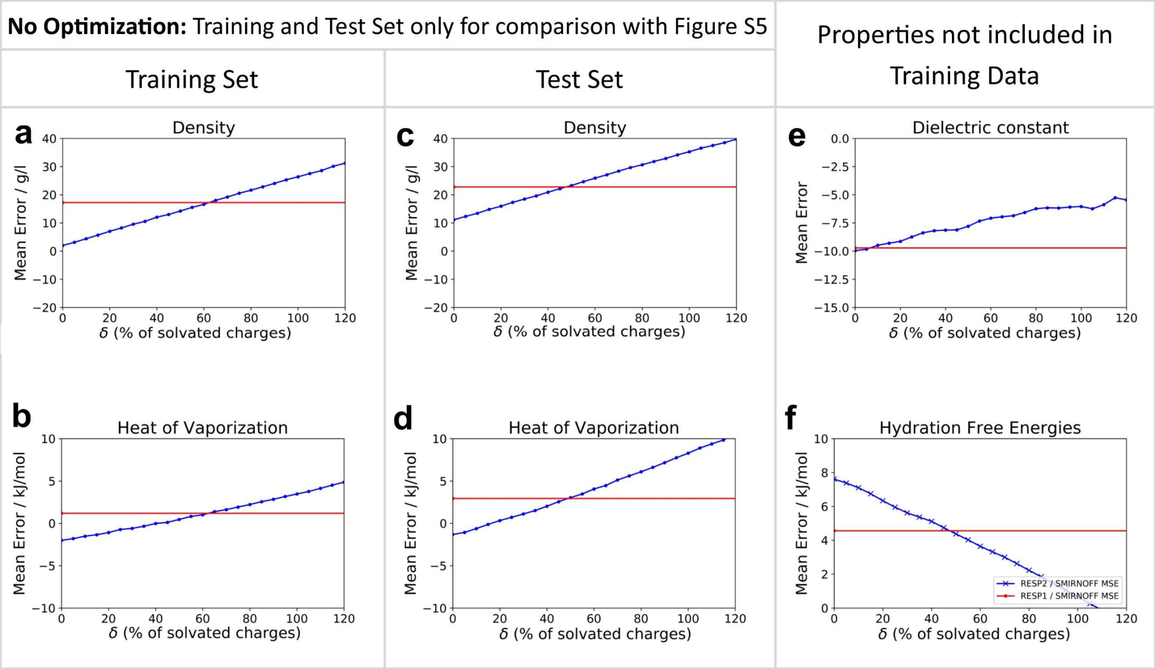

Supplementary Figure 4: Mean unsigned error with SMIRNOFF Lennard-Jones parameters: *Mean signed error of simulated results, relative to experiment, as a function of the charge mixing parameter δ with SMIRNOFF LJ parameters. No parameters are optimized for these results. Separation of training and test set is kept to make comparison with Figure S5 easier. Mean unsigned error for densities and HOV for the training-set (a,b) and test-set (c,d). Mean unsigned error for the dielectric constants (e) of the test-set and HFE error for all molecules in the FreeSolv database and either in the test or training-set (f). The red line are results obtained with RESP1 charges and is used as a reference.*

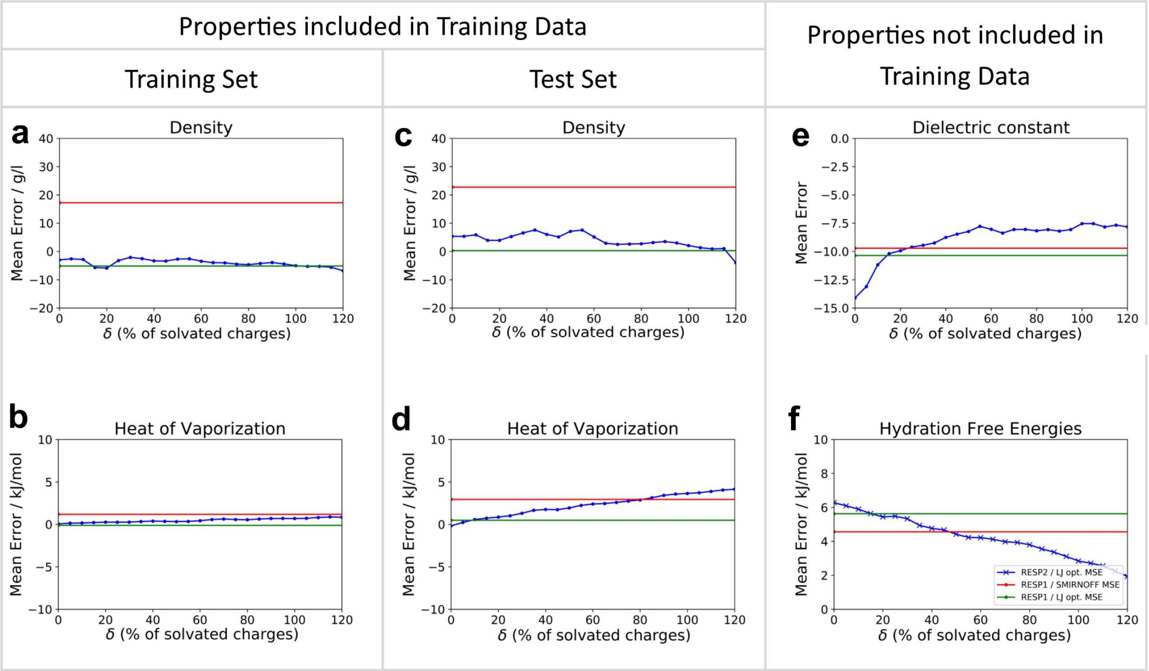

Supplementary Figure 5: Mean signed error with reoptimized Lennard-Jones parameters: *Mean signed error between theoretical and experimental results as a function of the charge mixing parameter δ with optimized LJ parameters. Mean unsigned error for densities and HOV for the training-set (a,b) and test-set (c,d). Mean unsigned error for the dielectric constants (e) for the test-set and HFE error for all molecules in the FreeSolv database and either in the test or training-set (f). The red line are results obtained with RESP1 charges and is used as a reference.*

8. Sample results with only one hydrogen LJ type

Supplementary Table 4: Mean unsigned errors obtained with LJ parameters trained in the context of $RESP2_{0.5}$ partial charges, with separate LJ types for polar and nonpolar hydrogens and for a single hydrogen type. Densities in g/l, heats of vaporization in kJ/mol

	**Polar and nonpolar H types**	**Single H type**
**Training set densities**	19.1	27.1
**Training set heats of vaporization**	2.28	4.75
**Test set densities**	37.7	38.7
**Test set heats of vaporization**	6.61	10.53

9. Optimized LJ parameters

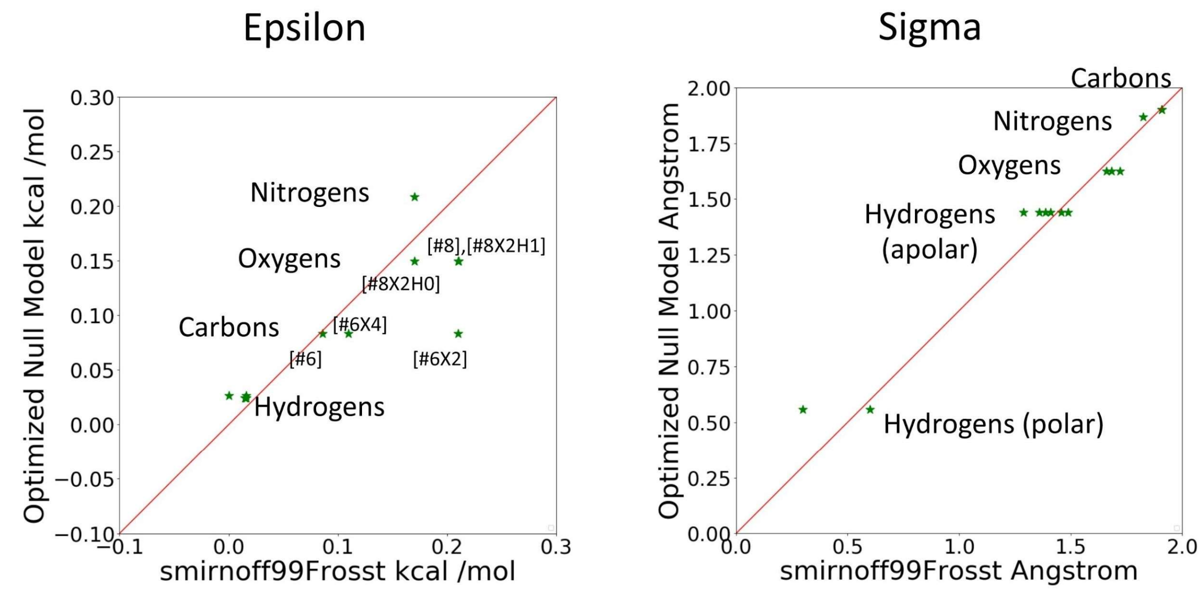

Supplementary Figure 6: Changes in LJ parameters: *Parameter changes for atoms in the test and training sets between SMIRNOFF and the reduced LJ set optimized with RESP2*$_{0.5}$. *Instead of seven hydrogen types, the reduced set distinguish between polar and apolar hydrogens. The three carbon terms are all reduced to one type, as well as the three oxygen types. For nitrogen, only one atom type exists in SMIRNOFF and is kept. Select SMIRNOFF LJ types are labelled with SMIRKS strings.*

Supplementary Note 2: Optimized LJ parameters for RESP2$_{\delta=0.5}$. Epsilon values in kcal/mol and rmin_half in Å.

<Atom smirks="[#1:1]" epsilon="2.442425552763e-02" id="n1" rmin_half="1.441450206517e+00" parameterize="rmin_half, epsilon"/>

<Atom smirks="[#6:1]" epsilon="8.316545253750e-02" id="n2" rmin_half="1.900104117994e+00" parameterize="rmin_half, epsilon"/>

<Atom smirks="[#8:1]" epsilon="1.494128902689e-01" id="n3" rmin_half="1.625860788705e+00" parameterize="rmin_half, epsilon"/>

<Atom smirks="[#7:1]" epsilon="2.085804532765e-01" id="n20" rmin_half="1.867010746380e+00" parameterize="rmin_half, epsilon"/>

<Atom smirks="[#1:1]-[#7,#8,#9,#16,#17,#35]" epsilon="2.658179674558e-02" id="n8" rmin_half="5.582920055246e-01" parameterize="rmin_half, epsilon"/>

Supplementary Note 3: Optimized LJ parameters for RESP2$_{\delta=0.6}$. Epsilon values in kcal/mol and rmin_half in Å.

<Atom smirks="[#1:1]" epsilon="2.276530034185e-02" id="n1" rmin_half="1.439694431063e+00" parameterize="rmin_half, epsilon"/>

<Atom smirks="[#6:1]" epsilon="8.400150760454e-02" id="n2" rmin_half="1.912583986068e+00" parameterize="rmin_half, epsilon"/>

<Atom smirks="[#8:1]" epsilon="1.504628783063e-01" id="n3" rmin_half="1.665073160695e+00" parameterize="rmin_half, epsilon"/>

<Atom smirks="[#7:1]" epsilon="2.061685252086e-01" id="n20" rmin_half="1.876266733552e+00" parameterize="rmin_half, epsilon"/>

<Atom smirks="[#1:1]-[#7,#8,#9,#16,#17,#35]" epsilon="2.672963104223e-02" id="n8" rmin_half="5.389607651519e-01" parameterize="rmin_half, epsilon"/>

Supplementary Note 4: Optimized LJ parameters for RESP1. Epsilon values in kcal/mol and rmin_half in Å.

<Atom smirks="[#1:1]" epsilon="2.007373778833e-02" id="n1" rmin_half="1.426322194675e+00" parameterize="rmin_half, epsilon"/>

<Atom smirks="[#6:1]" epsilon="8.285788790985e-02" id="n2" rmin_half="1.938260037810e+00" parameterize="rmin_half, epsilon"/>

<Atom smirks="[#8:1]" epsilon="1.668165991003e-01" id="n3" rmin_half="1.698013216722e+00" parameterize="rmin_half, epsilon"/>

<Atom smirks="[#7:1]" epsilon="1.811757801757e-01" id="n20" rmin_half="1.847472233860e+00" parameterize="rmin_half, epsilon"/>

<Atom smirks="[#1:1]-[#7,#8,#9,#16,#17,#35]" epsilon="7.437389444686e-03" id="n8" rmin_half="3.198481741167e-01" parameterize="rmin_half, epsilon"/>

All optimized FF files are also available on GitHub in a machine readable format.
